# Hair-Growth Potential of Ginseng and Its Major Metabolites: A Review on Its Molecular Mechanisms

**DOI:** 10.3390/ijms19092703

**Published:** 2018-09-11

**Authors:** Bu Young Choi

**Affiliations:** Department of Pharmaceutical Science and Engineering, Seowon University, Cheongju 28674, Korea; bychoi@seowon.ac.kr; Tel.: +82-43-299-8411; Fax: +82-43-299-8470

**Keywords:** ginseng, human-hair-follicle dermal papilla cells, WNT/β-catenin, Shh/Gli, TGF-β, BMP/Smad, mouse-hair growth

## Abstract

The functional aspect of scalp hair is not only to protect from solar radiation and heat/cold exposure but also to contribute to one’s appearance and personality. Progressive hair loss has a cosmetic and social impact. Hair undergoes three stages of hair cycle: the anagen, catagen, and telogen phases. Through cyclical loss and new-hair growth, the number of hairs remains relatively constant. A variety of factors, such as hormones, nutritional status, and exposure to radiations, environmental toxicants, and medications, may affect hair growth. Androgens are the most important of these factors that cause androgenic alopecia. Other forms of hair loss include immunogenic hair loss, that is, alopecia areata. Although a number of therapies, such as finasteride and minoxidil, are approved medications, and a few others (e.g., tofacitinib) are in progress, a wide variety of structurally diverse classes of phytochemicals, including those present in ginseng, have demonstrated hair growth-promoting effects in a large number of preclinical studies. The purpose of this review is to focus on the potential of ginseng and its metabolites on the prevention of hair loss and its underlying mechanisms.

## 1. Introduction

The hair-growth cycle comprises three distinct phases, the anagen, catagen, and telogen phases of independent hair follicles. Hair continues to grow during the anagen phase, followed by a transitional period of the catagen phase, which enters into the telogen phase, when hair is released from the follicle and falls. The anagen phase can be classified into a propagating anagen phase that involves the activation of new hair follicles, and an autonomous anagen phase, when hair growth and differentiation of hair follicles actively occur [1]. The normal hair-growth cycle is repeated about 20 times; however, it can be modified or shortened by internal or external factors such as hormones, stress, concurrent disease, exposure to environmental pollution, and smoking. Changes in the growth cycle leading to hair loss may be represented with the shortening of the anagen phase, premature ingression of the catagen phase, and the prolongation of the telogen phase. Early hair loss is medically termed as alopecia [2,3]. The number of people suffering from alopecia is increasing and approaching approximately 10 million throughout the world. Considering the pathological background of alopecia and its impact on an individual’s health and social value, there is now a growing interest in the development of novel therapeutics for its medical management. To date, the United States Food and Drug Administration (US-FDA) has approved two medications, minoxidil and finasteride, for the treatment of alopecia. Finasteride has been shown to prevent male pattern hair loss through the inhibition of type II 5α-reductase, which affects androgen metabolism. Although the exact mechanism of minoxidil has still not been elucidated, available research findings suggest that the hair-growth promotional effects of minoxidil are mediated through enhanced nutrient supply to hair follicles through vasodilation, opening of the K^+^ channel, and the activation of extracellular signal-regulated kinase (ERK) and protein kinase B (AKT/PKB) signaling, resulting in increased cell proliferation and inhibition of apoptosis in dermal papilla cells [4,5]. However, these drugs exhibit certain adverse effects, such as allergic contact dermatitis, erythema, and itching. While discontinuation of minoxidil leads to recurrence of alopecia, prolonged use of finasteride causes male sexual dysfunction and appears as a major cause of infertility and teratogenicity in females [6,7]. Thus, nontoxic chemicals with persistent hair-growth promoting effects have long been sought from the vast resources of natural products [8,9,10].

Ginseng is an ancient herbal remedy that was recorded in The Herbal Classic of the Divine Plowman, the oldest comprehensive Materia Medica, which was scripted approximately 2000 years ago. Contemporary science has revealed that ginseng contains a wide variety of bioactive constituents, especially a group of saponin compounds collectively known as ginsenosides, which are accredited with diverse biological activities, including the hair-growth potential of ginseng. Depending on the number of hydroxyl groups available for glycosylation via dehydration reactions, ginsenosides can be classified as protopanaxadiol (PPD) and protopanaxatriol (PPT). Common PPD-type ginsenosides include ginsenosides Rb1, Rb2, Rc, Rd, Rg3, F2, Rh2, compound K (cK), and PPD, whereas PPT-type ginsenosides include Re, Rf, Rg1, Rg2, F1, Rh1, and PPT [1]. Ginseng extract or its specific ginsenosides have been tested for their potential to promote hair growth. This review sheds light on the potential of ginseng and ginsenosides in promoting hair growth and delineating the mechanisms by which they function.

## 2. Biochemical Basis of Hair-Growth Promotion by Ginseng

**Figure 1:**
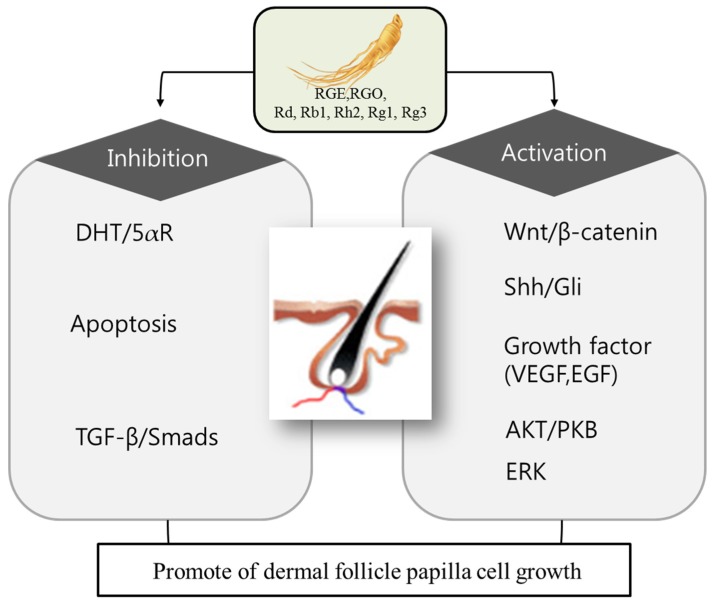
Potential molecular targets of ginseng in hair growth and loss. Ginseng exhibits therapeutic potential for hair growth and preventing hair loss by preventing the apoptosis of dermal follicle papilla cells. Ginseng components: RGE (red ginseng extract), RGO (red ginseng oil), ginsenoside Rd, Rb1, Rh2, Rg1, Rg3. antiandrogenic: DHT (dihydrotestosterone), 5-aR (5α-reductase). Apoptosis inhibition: TGF-β (transforming growth factor beta), Smads (homologues of the Drosophila protein, mothers against decapentaplegic (Mad) and the *Caenorhabditis elegan* sprotein Sma). Proliferation activation: WNT (wingless-type MMTV integration site family member), Shh (Sonic hedgehog), Gli (glioma-associated oncogene homolog), VEGF (vascular endothelial growth factor), EGF (epidermal growth factor), AKT/PKB (protein kinase B), ERK (extracellular-signal-regulated kinases).

There has been mounting evidence suggesting that ginseng and its major bioactive constituents, ginsenosides, promote hair growth by enhancing proliferation of dermal papilla and preventing hair loss via modulation of various cell-signaling pathways [11,12,13]. While the role of 5α-reductase enzyme in the hair-loss process has been well-documented [14,15], the emerging biochemical mechanisms of hair-follicle proliferation and the hair-loss process unravel new targets for designing novel therapeutics for the management of hair loss and alopecia (Figure 1). These targets include, but are not limited to, WNT/Dickkopf homologue 1 (DKK1), sonic hedgehog (Shh), vascular endothelial growth factor (VEGF), transforming growth factor-beta (TGF-β), matrix metalloproteinases (MMPs), extracellular signal-regulated protein kinase (ERK), and Janus-activated kinase (JAK). The following section summarizes the role of ginseng and its metabolites on hair growth.

### 2.1. Prevention of Radiation-Induced Skin Damage

Photoaging is one of the long-term effects of chronic sun exposure characterized by different inflammatory responses to ultraviolet radiation (UVR). Although exposure to solar UVR induces the synthesis of vitamin D, melanocortins, adrenocorticotropic hormone, and corticotropin- releasing hormone in human skin, and shows a beneficial effect, excessive UV irradiation is known to cause skin photodamage by inducing reactive oxygen species (ROS), precipitating skin inflammation, and promoting keratinocyte cell death. The impact of UVR exposure further leads to skin photoaging and carcinogenesis. However, the influence of UVR on skin appendages such as hair follicles is still in progress in many aspects. Accumulating evidence suggests that UVR exposure not only causes the damage of the hair shaft as an extracellular tissue, but also alters the hair-growth cycle by affecting keratinocyte and dermal papilla growth [16]. UV irradiation causes accumulation of ROS and activates MMPs, a class of tissue-degrading enzymes, thereby compromising dermal and epidermal structural integrity. Irradiation of normal human dermal papilla cells (nHDPC) with ultraviolet B (UVB) (≥50 mJ/cm^2^) exhibited ROS-mediated induction of apoptotic cell death [17]. Ginsenosides Rb2 [4] and 20 (S) PPD, but not 20 (R) PPD [4], have been reported to reduce the formation of ROS and MMP-2 secretion in cultured human keratinocytes (HaCaT) cells after exposure to UVB radiation. Likewise, ginsenoside Rg3 20 (S), but not 20 (R), reduced ROS generation in HaCaT cells and human dermal fibroblasts without affecting cell viability. The 20 (S) Rg3 also attenuated UVB-induced MMP-2 levels in HaCaT cells [6]. In another study, ginsenoside Rh2 epimers reduced UVB radiation-induced expression and activity of MMP-2 in HaCaT cells, but UVB-induced ROS formation was only suppressed by 20 (S)-Rh2 [7]. Because the extracellular matrix plays a critical role in hair-follicle function, degradation and matrix remodeling by MMPs affect the hair cycle [18,19]. The inhibitory effect of ginsenosides on UVB-induced activation of MMP2 suggests the potential of these ginseng saponins in hair-growth regulation.

Ginsenosides have also been shown to improve hair growth by attenuating radiation-induced cell death in the skin. Total-root saponins and ginsenoside Rb1 diminished apoptotic cells, as revealed by the accumulation of Ki-67-positive cells and elevated expression of Bcl-2, an antiapoptotic protein, in UVB-exposed human keratinocytes [20]. Ginsenoside F1, an enzymatically modified derivative of ginsenoside Rg1, also protected keratinocytes from radiation-induced apoptosis by maintaining a constant level of Bcl-2 and Brn-3a expression in UVB-irradiated HaCaT cells [21].

### 2.2. Antiaging Effects of Ginsenosides

Several studies have reported on the antiaging effects of various ginsenosides [22,23]. As a general outcome of antiaging effects, ginseng extract and ginsenosides maintain skin structural integrity and regulate hair-growth promotion. For instance, incubation of cultured human dermal fibroblasts with *Panax ginseng* for three days significantly increased cell proliferation and collagen synthesis [24]. The antiaging effects of *P. ginseng* root extract were attributed to the induction of type-1 pro-collagen via phosphorylation of Smad2 and activation of human collagen-A2 promoter in human dermal fibroblast. According to this study, *P. ginseng* root extract did not exhibit any sensitivity reaction to human skin [25]. Another marker of the aging process is wrinkle formation, which is often associated with a reduced level of hyaluronan in the dermis. Topical application of a major ginseng metabolite (compound K) on mouse skin elevated the expression of hyaluronan synthase-2, an enzyme that catalyzes the synthesis of hyaluronan, through Src kinase-dependent activation of ERK and AKT/PKB kinases in the dermis and papillary dermis of mice [26,27]. These antiaging effects result in improved skin health, thereby ensuring hair-follicle health and a regular hair cycle.

### 2.3. Modulation of TGF-β Signaling

The role of TGF-β in hair loss has been documented through the study revealing that treatment with a TGF-β antagonist can promote hair growth via preventing catagen progression [28]. Since TGF-β1 induces catagen in hair follicles and acts as a pathogenic mediator of androgenic alopecia [28], red ginseng extract can delay the catagen phase and holds the potential to promote hair growth. Administration of red ginseng extract at a dose of 20 or 60 mg/kg twice daily by gavage decreased TGF-β1 levels in UVB-irradiated mouse skin [29]. Likewise, topical administration of ginsenoside Re on to the back skin of nude mice for up to 45 days significantly increased hair-shaft length and hair existent time, and stimulated hair-shaft elongation in the ex vivo cultures of hair follicles isolated from C57BL/6 mouse. The hair-growth-promoting effects of ginsenoside Re were associated with the downregulation of TGF-β-pathway-related genes, which are involved in the control of hair-growth phase-transition-related signaling pathways [30]. It has been reported that brain-derived neurotrophic factor (BDNF) enhances transition from the anagen to the catagen phase through the activation of TGF-β [31]. Protopanaxatriol-type gisenoside Re promotes hair growth through the inhibition of TGF-β signaling pathways [30]. TGF-β-induced hair loss is associated with the hyperactivation of the c-Jun-N-terminal kinase (JNK) pathway [32]. The inhibition of JNK by Korean red ginseng has been attributed to the protective effects of ginseng on radiation-induced apoptosis of HaCaT cells [33].

Moreover, hair-follicle regression is partly regulated by the p75 neurotrophin receptor (p75NTR), which is a classical BDNF [34]. Since neurotrophins elicit their effects by interacting with high-affinity neurotrophin receptors, it would be a rational approach to develop neurotrophin-receptor antagonists as potential therapy for the treatment of hair loss, particularly androgenetic alopecia. A recent study has demonstrated that *P. ginseng* hexane extracts, which largely contain polyacetylenes, strongly inhibited β-nerve growth factor (β-NGF) interaction with p75NTR. Thus *P. ginseng*-derived polyacetylenes would be a potential therapeutic choice for the treatment of hair-growth disorders [35].

### 2.4. Inhibition of 5α-Reductase Enzyme

Progressive hair loss, also known as alopecia, occurs due to alternations in cell-signaling pathways in hair follicular cells resulting in the induction of apoptosis, changes in usual pattern of hair cycling and thinning, or fracture of the hair shaft. One of the major triggers for hair loss is the exposure to androgens, which in most cases are genetically predetermined among the individuals who have androgenic alopecia. The androgen that mainly plays a role in altering hair cycling is 5α-dihydrotestosterone (DHT), which is a metabolite of testosterone. The conversion of testosterone to DHT is mediated by the 5α-reductase (5αR) enzyme in each follicle [27]. Treatment with 5α-reductase inhibitors, e.g., finasteride, prevents the development of alopecia and increases scalp-hair growth. In several in vivo experiments, topical application of ginseng extract or ginsenosides was reported to enhance hair growth. Rhizomes of *P. ginseng* (red ginseng) containing a considerable amount of ginsenoside Ro inhibited the activity of 5α-reductase. Ginsenoside Rg3 and Rd also exhibited similar inhibitory effects on this enzyme [36]. The inhibition of 5αR enzyme activity was more pronounced with extracts of red-ginseng rhizomes as compared to that of ginseng main-root extract. Ginsenosides Ro derived from rhizome extract and ginsenoside Rg3 obtained from main-root extract attenuated the 5αR enzyme activity with IC_50_ values of 259.4 and 86.1 µm, respectively. Another variety of ginseng, the *Parribacus japonicas* rhizome extract that contains a larger quantity of ginsenoside Ro also inhibited 5αR enzyme activity. Topical administration of red-ginseng rhizome extracts (2 mg/mouse) and ginsenoside Ro (0.2 mg/mouse) onto shaved skin of C57BL/6 mice abrogated testosterone-mediated suppression of hair regrowth [36].

### 2.5. Modulation of Wnt/Dickkopf Homologue 1 (DKK1) Signaling

Wingless-type integration-site (WNT) signaling plays a key role in hair-follicle development. The blockade of Wnt signaling by overexpression of the WNT inhibitor, DKK1, prevents hair-follicle formation in mice [37]. β-catenin signaling is essential for epithelial stem-cell fate since keratinocytes adopt an epidermal fate in the absence of β-catenin [38]. Treatment with ginsenoside F2 resulted in a 30% increase in the proliferation of HHDPC and HaCaT cells as compared to that of finasteride. Ginsenoside F2 increased the expression of β-catenin and its transcriptional coactivator Lef-1, while it decreased the expression of DKK-1 in HHDPC as well as in the skin of C57BL/6. Administration of ginsenoside F2 promoted hair growth as compared to finasteride, as revealed by an increase in the number of hair follicles, thickness of the epidermis, and follicles of the anagen phase, suggesting that F2 induces the anagen phase and stimulates hair growth through the modulation of the Wnt signal pathway [39]. In another study by Matsuda et al., the hair growth-stimulating activity of the methanol extract of red ginseng in an organ culture of mouse vibrissal follicles was attributable to ginsenosides Rg3 and Rb1 [40]. Treatment of cultured outer root sheath (ORS) keratinocytes with *P. ginseng* extract in the presence or absence of DKK-1 has revealed that *P. ginseng*-extract treatment increased the Bcl-2 to Bax ratio, and the anagen to catagen ratio, and reversed DKK-1-mediated suppression of the Bcl-2/Bax ratio. *P. ginseng* extract antagonizes DKK-1-induced catagen-like changes, in part, through the regulation of apoptosis-related gene expression in hair follicles [41].

### 2.6. Modulation of Sonic Hedgehog (Shh) Signaling

Shh/Gli (glioma-associated oncogene homolog) regulates hair-follicle development during embryonic life and influences the cycling and growth of hair follicles in adults by promoting telogen-to-anagen transition of follicular cells and epidermal growth [42,43,44]. Mice harboring the mutant form of Shh have small dermal papillae characterized by the presence of abnormal hair follicular cells that are incapable of maintaining normal hair morphogenesis [44]. Attenuation of Shh activity by a monoclonal antibody targeting Shh diminished hair growth in mice, indicating the importance of Shh signaling in hair-growth promotion [45]. Treatment with red-ginseng oil reversed testosterone-induced suppression of hair regeneration in C57BL/6 mice by increasing the expression of Shh/Gli pathway-related proteins, including Shh, Smoothened (Smo), and Gli1. Additionally, two major compounds in red-ginseng oil, linoleic acid and β-sitosterol, were also found to activate the Shh/Gli signaling pathway in testosterone-treated mice. Topical application of bicycle (10.1.0) tridec-1-ene was unlikely to significantly accelerate protein levels of Shh and Gli1, but likely to increase Smo expression [46].

### 2.7. Modulation of JAK2-STAT3 Signaling

Cytokines, such as interleukins (ILs) and interferons (IFN), are inflammatory-signaling molecules that, upon overexpression and/or secretion, cause skin inflammation. Hair follicles are usually immune-tolerated areas, where natural killer (NK) cells remain suppressed [47]. Such immune activation is supported by the presence of CD8+ T cells and NKG2D+ cells around the peribulbar area of the affected hair follicles [48] and upregulation of several ILs, such as IL-2, IL-7, IL-15, and IL-21, and IFN-γ [49]. Loss of immune tolerance or immune activation, due to the upregulation of major histocompatibility complex (MHC class I) or UL16-binding protein 3 (ULBP3) molecules, leads to the activation of a cytotoxic cluster of differentiation 8-positive (CD8+) and NK group 2D-positive (NKG2D+) T cells to the hair follicles [50,51], thereby leading to hair-follicle dystrophy and acceleration of the catagen phase [52]. Since JAK/Signal transducer and activator of transcription-3 (STAT3) pathway plays a critical role in mediating the activation of CD8+ NKG2D+ T cells, the inhibition of JAK appears as a plausible target for developing a therapy for hair loss [49]. In fact, a number of JAK inhibitors, such as tofacitinib and roxulitinib, are in the progress of developing a therapy for alopecia [53]. Ginsenoside Rk1 inhibited the lipopolysaccharide- stimulated phosphorylation of JAK2 and STAT3 in murine macrophage cells [54]. It would be interesting to investigate whether ginsenoside Rk1 or other ginsenosides can target JAK2 signaling in dermal papilla and diminish activation of NKG2D+ T cells. Moreover, the pathogenesis of alopecia areata is believed to involve inflammatory cytokines IL-17A and monoclonal antibodies against IL-17A secukinumab-caused hair regrowth in human volunteers [55]. Treatment of Th17 cells with *Panax notoginseng* saponins diminished the proliferation and differentiation of Th17 cells and decreased IL-17 expression [56]. Topical application of ginsenoside F2 also ameliorated phorbol ester-induced dermal inflammation by inhibiting the production of IL-17 and ROS in γδT cells and neutrophils, respectively, in mouse-ear skin [57]. These findings suggest that ginsenosides may enhance hair growth in alopecia areata by regulating IL-17 secretion. 

### 2.8. Activation of Dermal Papillary Cell Proliferation 

Various intracellular signaling molecules, including kinases and growth factors, play a critical role in stimulating hair growth by promoting dermal papillary-cell proliferation. VEGF, which is released from the epithelium, is a signaling protein that increases the vascular network surrounding the hair follicle [58]. Ginsenoside Rg3 promotes hair growth by upregulating VEGF expression [36]. Shin et al. also demonstrated that Rg3 increased the proliferation of human dermal papillary cells, which was associated with elevating the mRNA level of VEGF. In mouse-hair follicles in vivo, Rg3 not only increased the expression of VEGF but also stimulated stem cells by upregulating factor-activating CD34, and promoted hair growth even more than minoxidil [39].

Signaling pathway ERK, usually activated by mitogens, plays an important role in the proliferation of human hair-follicle dermal papillary cells (HHDPCs) [59]. Both red-ginseng extract (RGE) and ginsenoside-Rb1 activated the ERK signaling pathway. Thus, the proliferation of HHDPCs by red ginseng may be mediated by the ERK signaling pathways [12]. Another intercellular kinase, AKT/PKB, transmits critical signals for cell survival, and also regulates the survival of dermal papillary cells (DPCs) as an antiapoptotic molecule [60]. Therefore, the activation of AKT/PKB by red-ginseng extract and ginsenoside-Rb1 may prolong the survival of HHDPCs [12].

The Bcl-2 family proteins consists of more than a dozen members, which are either antiapoptotic or proapoptotic in nature [61]. During the hair cycle, the DPC is the only region where Bcl-2 is expressed consistently and is considered to resist apoptosis [62]. *Fructus panax ginseng* extract increases the expression of Bcl-2 but decreases Bax expression, a proapoptotic species, in cultured DPCs [63].

## 3. Evidence from In Vivo Animal Studies

Ginsenosides Rb1 and Rd from *P. ginseng* also exert a stimulating effect on hair follicles, and thus, appear as potential therapeutic agents. One suggested mechanism for this effect of ginsenosides Rb1 and Rd is the induction of p63 [64]. Topical application of *P. ginseng* extract (2.5%) failed to stimulate hair growth as compared to minoxidil in athymic nude mice [8]. The lack of the hair growth-promoting effect of ginseng in this study compared to other herbal products exhibiting hair growth may not be appropriately judged, as the nude mice are basically hairless or have limited fine hairs with poorly defined hair cycles. However, application of *P. ginseng* extract by intraperitoneal or per oral prior to gamma irradiation to adult N:GP mice diminished apoptosis and promoted hair medullary-cell repair [65]. In another study, C57BL/6 mice were subjected to treatment with ginsenoside F2 or finasteride. As compared to the finasteride-treated group, the ginsenoside F2-treated group showed 20% higher hair-growth rates as evidenced by increased number of hair follicles, epidermal thickness, and proportion of follicles in the anagen phase. This hair-growth promoting effect of ginsenoside F2 was mediated, at least in part, through the activation of the Wnt-β-catenin pathway via blockade of Dkk [39]. Truong and colleagues [46] also reported that hair-regenerative capacity was significantly restored by treatment of red-ginseng oil and its major compounds in testosterone-treated mice.

## 4. Human Clinical Studies

Although individual ginsenosides are yet to be investigated for hair-growth promotion in human clinical trials, there have been few interesting human studies documenting the potential of Korean red ginseng in hair-growth promotion. Oh et al. studied hair-growth efficacy and safety of Korean red ginseng (KRG) in alopecia areata (AA), a model of androgenic alopecia, in human subjects. According to this study, human volunteers were treated with corticosteroid intralesional injection (ILI) with or without treatment with KRG. Hair growth in both the ILI-alone and ILI-plus-KRG patient group was monitored using Folliscope 2.5 for 12 weeks. Average hair density and hair thickness were significantly increased upon addition of KRG with ILI, suggesting that KRG may be considered as a useful complimentary food for gaining efficacy in the treatment for AA [66]. Kim et al. reported the effectiveness of Korean red ginseng in increasing the thickness and density of hair in human volunteers [11]. Moreover, combination treatment with topical minoxidil and oral KRG is more effective than topical minoxidil treatment alone for promoting hair growth. Therefore, KRG is expected to be a helpful supplement in the treatment of hair loss [67]. Keum et al. examined the potential of KRG in preventing premature hair-follicle dystrophy using a human hair-follicle organ-culture model. According to this study, human occipital scalp-skin specimens were obtained from patients undergoing hair-transplantation surgery, and follicular keratinocytes cells (FKC) were cultured in vitro. Treatment of FKCs with 4-hydroxycyclophosphamide (4-HC), a metabolite of chemotherapeutic agent cyclophosphamide, attenuated human hair growth, induced premature catagen development, diminished proliferation, and stimulated apoptosis of hair matrix keratinocytes. Pretreatment with KRG protected against 4-HC-induced hair-growth inhibition and premature catagen development partly by blocking 4-HC-induced p53 and Bax/Bcl2 expression [13].

## 5. Conclusions

The use of plant products in therapy has long been practiced and has appeared to be generally safe. Ginseng is a multipurpose natural medicine with a long history of medical application throughout the world, particularly in Eastern countries. The medical use of ginseng is not only restricted to the improvement of general wellness, but also extended to the treatment of organ-specific pathological conditions. In the field of dermatology, ginseng and ginsenosides have been shown to regulate the expression and activity of major proteins involved in hair-cycling phases. The promotion of hair growth and prevention of hair loss by ginseng and its metabolites are associated with the induction of anagen and delaying of catagen phases. Although the underlying mechanisms by which ginseng and its metabolites regulate hair cycling have been explored to a limited extent, further studies, especially focusing on extended human trials, are required to establish this natural remedy for hair loss. Alopecia, originating from a variety of causes, including hyperactivation of androgenic signals, exposure to chemotherapeutics, aging, or skin photodamage, is considered as a skin pathology and has great psychosocial impact. Thus, it would be a plausible approach to develop hair growth-stimulating formulations, either as FDA-approved therapeutics or as cosmeceuticals, by using the index component of red ginseng (Table 1).

## Figures and Tables

**Table 1:** Molecular mechanisms underlying hair-proliferative and antiapoptosis-inducing activity of ginseng.

Type	Study Model	Dosage	Action Mechanism	Target	Reference
Fructus panax	Human hair dermal papilla cells	0.8, 4, 20, 100, 500 μg/mL	FPG elicited the proliferation of DPC by the upregulation of antiapoptotic Bcl-2 accompanied by the inhibition of apoptotic Bax expression	Apoptosis	[4]
ginseng extract (FPG) (95% EtOH)	Male six-week-old C57BL/6 mice	1 mg/mL			
Ginsenoside Re	Male six-week-old C57BL/6 mice	1 or 5 mg/d	Ginsenoside Re is the effective constituent in Panax ginseng that promotes hair growth through inhibition of transition related TGF-β signaling pathways.	TGF	[5]
	Cultured C57BL/6 mouse HFs	10 or 50 mg/L			
	HeLa cells	10 mg/L			
Polyacetylenes isolated from *P. ginseng*	Neurotrophin receptor-binding inhibition assay	sample solution (10, 30, and 100 μM)	Inhibits BDNF-TrkB binding.	Growth	[6]
Ginsenoside F2	Human hair dermal papilla cells	0.01, 0.1, 1, and 10 μM	(1) Stimulates proliferation of HHDPC and HaCaT; (2) increases β-catenin and Lef-1 expression and decreases DKK-1 expression in HHDPC; (3) hair anagen induction and acceleration of hair growth in mouse model; (4) increases β-catenin expression and decreases DKK-1 expression in mouse tissue.	WNT	[7]
	Human keratinocyte (HaCaT) cells				
	Male six-week-old C57BL/6 mice	0. 5 and 2.5 mg/kg			
Root of PG extract (70% EtOH)	Human ORS keratinocytes	20 ppm	PG extract may enhance ORS and hDPC stimulation of hair follicle growth despite the presence of DKK-1, a strong catagen inducer	WNT	[29]
	Anagen HFs from human scalp-skin specimens				
Korean Red Ginseng (KRG)	Human (patients diagnosed with AA)	Treated with corticosteroid ILI while taking KRG	KRG can result in improved hair regrowth in AA patients.		[20]
KRG extract	Follicular keratinocytes (FKCs)	0~1000 μg/mL	KRG may protect against 4-HC-induced premature dystrophy as it occurs in CIA in vivo. Possible mechanisms include the stimulation of hair matrix keratinocyte proliferation and inhibition of hair matrix keratinocyte apoptosis, which are possibly mediated through modulation of p53 and Bax/Bcl-2 expression.	Apotosis	[21]
	Human anagen hair follicles	500 μg/mL			
Red ginseng extract (RGE)	Six-week-old female C57BL/6 mice	3%	RGE and its ginsenosides may enhance hDPC proliferation, activate the ERK and AKT/PKB signaling pathways in hDPCs, upregulate hair matrix keratinocyte proliferation, and inhibit DHT-induced androgen receptor transcription.	Growth 5aR	[16]
RGE, insenoside-Rb1	Human hair follicles	100 μg/mL			
Red ginseng oil (RGO)	C57BL/6 mice	RGO 10%	Upregulates Wnt/-catenin and Shh/Gli pathways-mediated expression of genes such as β-catenin, Lef-1, Sonic hedgehog, Smoothened, Gli-1, Cyclin D1, and Cyclin E in TES-treated mice. RGO and its major components reduce the protein level of TGF-β but enhance the expression of antiapoptotic protein Bcl-2.	WNT Shh Growth TGF Apoptosis	[25]
KRG	Human (patients with female pattern hair loss)	Oral	Patients about the size of the vertex spot, hair loss on the top of scalp, bitemporal recession, hair shedding, hair quality, and overall satisfaction; group 2 was more satisfied at 24 weeks.		[68]
Ginsenoside Rg3	Human DP cells	1, 5, 10 μM	Dose-dependent increases in VEGF, CD8, CD34 Rg3 might increase hair growth through stimulation of hair-follicle stem cells	Growth	[69]
	Female C57BL/6 mice	1000 μM			
Red ginseng (RGE)	B6C3F1 mice	20, 50 μg/mL	Hair growth-promoting assay using mouse vibrissal follicles in organ culture	Growth	[26]
White ginseng (WGE) Ginsenoside-Rb1 (G-Rb1), Rg1 (G-Rg1), -Ro (G-Ro)	Mouse vibrissal hair follicles	10 μg/mL			
Ginseng rhizome Ginsenoside Ro	C57BL/6 mice	extracts of red ginseng rhizomes (2 mg/mouse) and ginsenoside Ro (0.2 mg/mouse)	Inhibitory activity against 5αR in the androgenetic alopecia model.	5aR	[27]
Ginsenosides Rb1, Re, and Rg1	Cultured hHFs	2, 5, and 10 mg/mL PG extracts and 1 mM of the ginsenosides Rb1, Re and Rg1	PG extract using hHF organ culture, and promoting hair growth through similar mechanisms to those of minoxidil.	5aR	[36]
